# Critical Assessment of Curvature-Driven Surface Hopping Algorithms

**DOI:** 10.1021/acs.jctc.5c01176

**Published:** 2025-09-16

**Authors:** Tomáš Jíra, Jiří Janoš, Petr Slavíček

**Affiliations:** Department of Physical Chemistry, 52735 University of Chemistry and Technology, Technická 5, Prague 6 16628, Czech Republic

## Abstract

Trajectory surface-hopping (TSH) methods have become the most used approach in nonadiabatic molecular dynamics. The increasingly popular curvature-driven schemes represent a subset of TSH based on the implicit local diabatization of potential energy surfaces. Their appeal partly stems from compatibility with machine-learning frameworks that often provide only local PES information. Here, we critically assess the limitations of these curvature-based algorithms by examining three challenging scenarios: (i) dynamics involving more than two strongly coupled electronic states; (ii) trivial crossings; and (iii) spurious transitions arising from small discontinuities in multireference potential energy surfaces. Furthermore, we extend the Landau–Zener surface hopping (LZSH) method beyond two-state systems and introduce practical modifications to enhance its robustness. The performance is benchmarked on both low- and higher-dimensional model Hamiltonians, as well as realistic molecular systems treated with *ab initio* methods. While curvature-driven TSH using the explicit electronic coefficient propagation qualitatively captures the dynamics in most cases, we find no regime where it outperforms LZSH, especially when trivial crossings, multistate crossings, or discontinuities are encountered. Hence, we advocate for using a conceptually simple but solid LZSH method when nonadiabatic couplings are not available.

## Graphical abstract

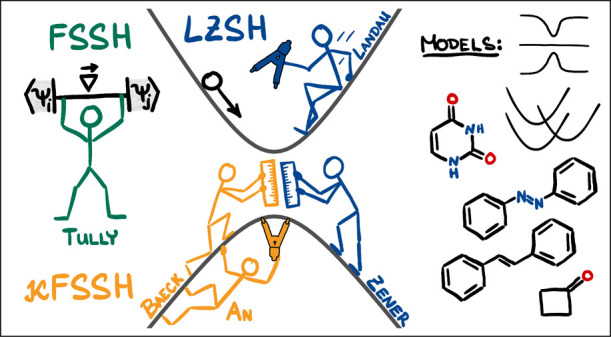

## 1 Introduction

Molecular dynamics has proven to be a powerful tool for quantitatively modeling chemical reactions.^1^ For photochemical systems, however, simulations must typically go beyond the adiabatic approximation and account for coupled electronic and nuclear motion.^2^ The dynamics in such cases appear on multiple potential energy surfaces (PESs), with coupling treated beyond perturbation theory.

With suitably chosen model potentials, highly accurate quantum approaches such as the multiconfigurational time-dependent Hartree (MCTDH) method can be employed.^3,4^ More often, however, we study reactions without detailed prior knowledge, relying instead on on-the-fly electronic-structure data.^5,6^ As these data are local, trajectory-based methods incorporating surface hopping between electronic states have become standard for simulating nonadiabatic dynamics, beginning with the pioneering *ab initio* studies.^2,7,8^ These methods integrate seamlessly with *ab initio* codes, offer intuitive interpretations of dynamics, and are computationally efficient and trivially parallelizable.

A key distinction between surface hopping schemes lies in how electronic transitions are modeled. For many years, the field was dominated by the Landau–Zener–Stückelberg–Majorana framework (here simply referred to as Landau–Zener, or LZ).^9–13^ This formalism assumes two-state crossings with linear (diabatic) potentials, constant coupling and constant velocity, yielding reliable predictions when only two states are coupled. It has proven valuable in both qualitative and quantitative contexts, particularly in charge-transfer reactions.^14^ Notably, the LZ model was also employed in the first molecular dynamics surface hopping treatments.^15^ To address the limitations of the LZ approach in multistate systems, Tully introduced the fewest-switches surface hopping (FSSH) algorithm.^16^ Designed for general potential surfaces without localizing the crossing region, FSSH gained popularity alongside advances in electronic structure methods and quickly became a standard in nonadiabatic dynamics.^17^

Yet several issues persist. Chief among them is the lack of electronic decoherence in FSSH, typically handled through *ad hoc* corrections,^18,19^ though more principled approaches have emerged.^20–23^ FSSH also struggles with trivial crossings (degenerate diabatic states with negligible coupling) common e.g., in molecular crystals.^24–27^ Moreover, the algorithm is often implemented—due to its simplicity—using nonadiabatic coupling vectors (NACVs), which are often divergent or unavailable. As an alternative, some implementations directly approximate the time derivative coupling (TDC) via electronic wave function overlaps,^28,29^ enhancing FSSH stability.

These limitations have renewed interest in alternative, often more pragmatic, hopping schemes.^30–36^ This trend reflects a growing consensus that the choice of electronic structure method more strongly determines simulation outcomes than the hopping algorithm itself.^37–44^ Consequently, robustness and computational feasibility are increasingly prioritized. While simple schemes that switch states upon encountering a new surface are sometimes used,^15^ more physical approaches revisit the two-state problem. For instance, the mapping approach to surface hopping (MASH) addresses decoherence rigorously for two-state systems,^20^ and Landau–Zener surface hopping (LZSH) has been reformulated in the adiabatic representation.^45^ The latter removes the need for NACVs and allows a natural treatment of intersystem crossings, which is used in numerous studies.^31–33,46,47^ More recently, Baeck and An proposed an approximation to nonadiabatic couplings based on PES curvature,^48^ leading to curvature-driven fewest-switches schemes that allow transitions throughout a trajectory.^30,40,49–51^ These methods can be easily combined with any electronic structure code capable of delivering potential energy surfaces, without the need to explicitly calculate the nonadiabatic couplings.

The present work focuses on curvature-based approaches derived under the two-state assumption. This includes both the LZSH and curvature-driven fewest switches surface hopping methods. We aim to identify regimes where such methods fail in photodynamics simulations. We consider three critical challenges: (i) three-state interactions, where methods designed for two states may misrepresent couplings, (ii) trivial crossings, which occur frequently in condensed phase systems and are known to cause failures in conventional hopping methods,^52,53^ and (iii) slight discontinuities in PESs, common in *ab initio* surfaces, which may degrade curvature-based schemes.

The remainder of this article is organized as follows. Section 2 introduces the theoretical framework and algorithms. Section 3 provides computational details for all the calculations. Section 4.1 presents benchmark results on model potentials designed to stress-test the methods. Section 3.2 showcases the surface hopping algorithms on a well-known vibronic coupling model of the uracil cation. Sections 4.3 and 4.4 extend this analysis to realistic molecular systems, focusing on the effects of PES continuity.

## 2 Theory

### 2.1 Trajectory Surface Hopping

In this section, we outline the theoretical framework of trajectory surface hopping (TSH). The key idea underlying TSH is that the nuclei are treated classically and propagated via Newton’s equations of motion, whereas the electrons are treated quantum mechanically. Under this approximation, the nuclei evolve on a potential energy surface obtained using the electronic time-independent Schrödinger equation.

The electronic wave function Φ­(**r**, *t*;**R**(*t*)) is then expanded as a linear combination of adiabatic eigenstates ϕ_*i*_(*r*;**R**(*t*)) at nuclear position **R**(*t*) of the electronic Hamiltonian as

$$\Phi (r,t;R(t))=\sum_{k}c_{k}(t)\phi_{k}(r;R(t))$$ 1

where the time-dependent coefficients *c*_*k*_(*t*) carry information about the population and phase of each electronic state ϕ_*k*_. For simplicity, we will omit the explicit dependence of the wave functions on their dynamical variables in subsequent equations. To obtain the equations of motion for the coefficients *c*_*k*_, we substitute eq 1 into the time-dependent Schrödinger equation (TDSE), obtaining

$$i\hbar \frac{dc_{j}}{dt}=\sum_{k}c_{k}\left(H_{jk}^{\mathrm{el}}-i\hbar \left\langle \phi_{j}|\frac{\partial \phi_{k}}{\partial t}\right\rangle \right)$$ 2

where the term $\sigma_{jk}=\left\langle \phi_{j}|\frac{\partial \phi_{k}}{\partial t}\right\rangle$ is the time derivative coupling. In the adiabatic basis, the electronic Hamiltonian **H** ^el^ is diagonal, so the TDC alone governs the transfer of population between adiabatic states. Because quantum chemical calculations can provide NACVs, $d_{jk}=\left\langle \phi_{j}|\frac{\partial \phi_{k}}{\partial R}\right\rangle$ , instead of time derivative couplings, we can employ the chain rule to write

$$\sigma_{jk}=\left\langle \phi_{j}|\frac{\partial \phi_{k}}{\partial t}\right\rangle =\frac{dR}{dt}\cdot \left\langle \phi_{j}|\frac{\partial \phi_{k}}{\partial R}\right\rangle =v\cdot d_{jk}$$ 3

With this manipulation, we propagate the nuclei together with the electronic wave function on a single adiabatic surface, relying only on electronic gradients and NACVs. The TSH algorithm needs to be supplemented by a procedure to allow transitions (hops) between different (adiabatic) surfaces. Several methods are used, including a simple approach in which hops occur whenever the energy gap between adiabatic surfaces appears below a preset threshold.^54^ Methods used within this document are described in the following sections.

### 2.2 Landau–Zener Surface Hopping

The Landau–Zener surface hopping algorithm, originally developed nearly a century ago, provides a remarkably straightforward and powerful framework for modeling nonadiabatic transitions.^9–11^ In the LZSH framework, an analytic solution of TDSE in eq 2 for a simplified model is applied throughout the dynamics. Hence, the electronic TDSE is not propagated along trajectories. Consider a two-level system described by the diabatic Hamiltonian representing the crossing between potential energy surfaces

$$H=\left[\begin{array}{cc} -\alpha t & H_{12}\\ H_{21} & \alpha t \end{array}\right]$$ 4

where α is an arbitrary constant and the diabatic coupling *H*_12_ is independent of time. The corresponding TDSE (eq 2 in diabatic basis) is written as

$$i\hbar \frac{d}{dt}\left[\begin{array}{c} c_{1}(t)\\ c_{2}(t) \end{array}\right]=H\left[\begin{array}{c} c_{1}(t)\\ c_{2}(t) \end{array}\right]$$ 5

with *c*_1_ and *c*_2_ representing the coefficients of the diabatic wave function. Under the initial condition *c*_1_(*t* → –*∞*) = 1, an exact solution of eq 5 leads to

$$c_{1}(t\to \infty )=\exp\left(-\pi \frac{H_{12}^{2}}{\alpha }\right)$$ 6

The hopping probability from state 1 to state 2 then reads^55,56^

$$P_{1\to 2}^{\mathrm{LZ},\mathrm{dia}}={\left|c_{2}(t\to \infty )\right|}^{2}=1-{\left|c_{1}(t\to \infty )\right|}^{2}=1-\exp\left(-2\pi \frac{H_{12}^{2}}{\alpha }\right)$$ 7

After simple algebraic manipulation and switching to general electronic state indexes, the adiabatic transition probability is obtained as^45^

$$P_{i\to k}^{\mathrm{LZ}}=\exp\left(-\frac{\pi }{2\hbar }\sqrt{\frac{Z_{ik}^{3}}{{\mathrm{Z̈}}_{ik}}}\right)$$ 8

where *Z*_*ik*_ = |*E*_*k*_ – *E*_*i*_| is the energy difference between states with *E*_*i*_ and *E*_*k*_ being the energies of adiabatic electronic states *i* and *j* at the current nuclear geometry. This probability is evaluated upon encountering the minimum of the energy difference.

It is important to note that the LZSH algorithm is intrinsically limited by its assumptions. It is formulated solely for *two-state systems* with *linearly time-dependent energies* and *constant diabatic coupling*. Although this constitutes a considerable approximation, the method provides a basis for our qualitative understanding of nonadiabatic processes and often yields quantitative results that are in reasonable agreement with those obtained using the FSSH approach.^32,37,57^

Since the LZSH algorithm is defined only for two-state crossings, we have extended the algorithm for multistate systems. When a multistate crossing is identified, our modified LZSH algorithm computes the transition probabilities from the current state only to a state for which *Z̈*_*ik*_ has a maximum value. The motivation behind the scheme stems from the fact that the two interacting states will exhibit the largest second derivatives, while the noninteracting states are expected to have relatively smaller second derivatives. We will refer to this algorithm as LZSH_maxcurv_. We also inspect a variant of LZSH, where we only calculate the transition probability to the energetically closest state. This modification will be referred to as LZSH_nearest_. The two versions of multistate LZSH are schematically depicted in Figure 1. Moreover, we investigated a version where we allow hops only to the state with maximum hopping probability, but it provides the same results as the LZSH_nearest_ algorithm. Hence, we do not discuss it further. Note that for a two-state system, all of these multistate variants reduce to the original LZSH scheme.

**Figure 1:**
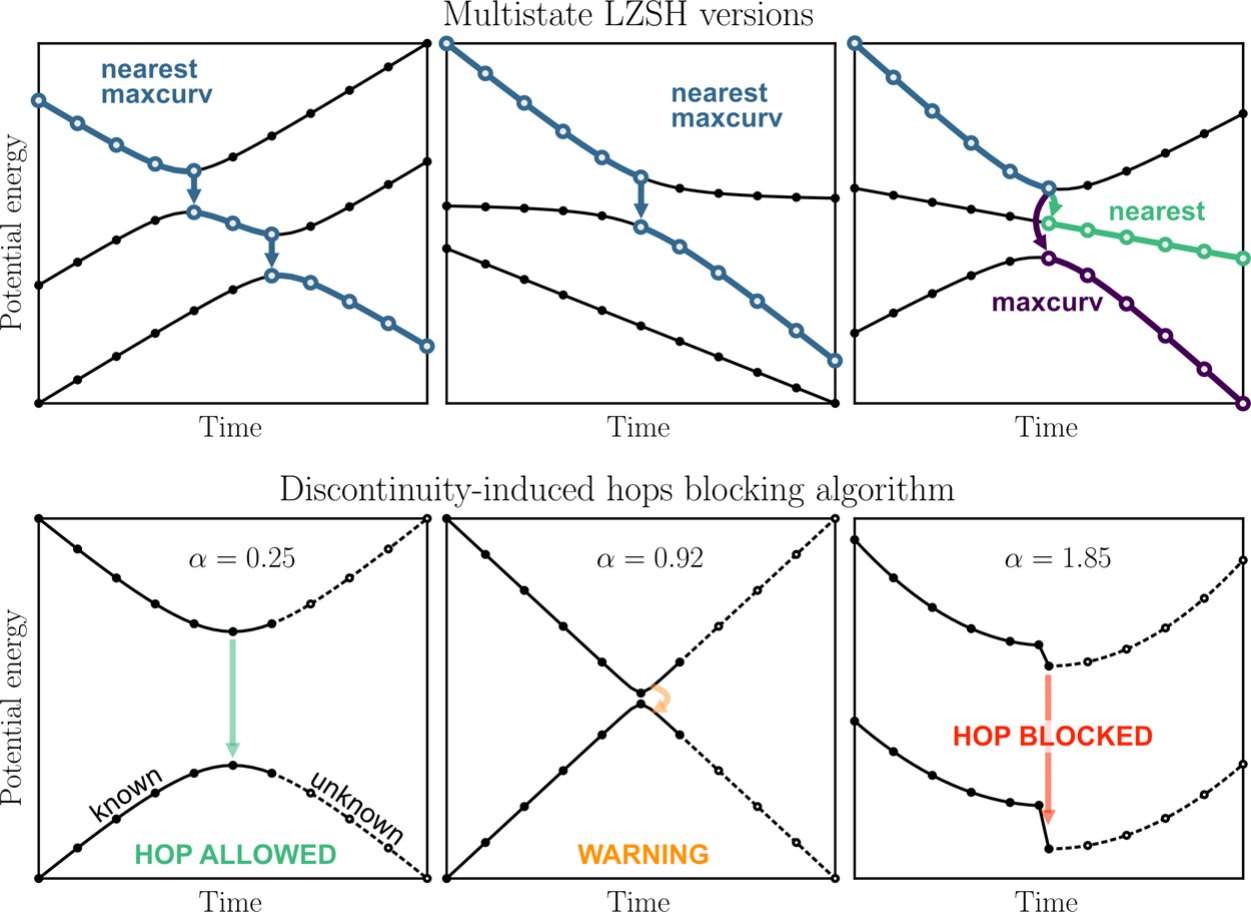
Top row: Schematic trajectories for typical situations encountered in photodynamics depicting principles of the two multistate LZSH variants. While both schemes are identical when only the neighboring states are interacting, they differ if the interacting states encompass a noninteracting state. Bottom row: Illustration of the performance of the blocking algorithm for discontinuity-induced hops in three different situations encountered in typical trajectories. For typical avoided crossing (left), the α value is small, and the algorithm allows all hops. If a very sharp conical intersection is encountered (middle), values of α around one are typical. In such a situation, the algorithm is not able to recognize from the known data whether a conical intersection or a discontinuity was encountered. Hence, a warning is issued. If the coefficient α exceeds 1.3 (situation on the right), a discontinuity almost certainly occurred, and the hop is blocked.

An alternative multistate extension of the Landau–Zener model has been proposed by Smith and Akimov, in which no final state is favored but the hopping probabilities are rather normalized accordingly.^58^ We deliberately chose not to employ this variant, as it yields incorrect results for our specific three-state model systems. Smith and Akimov further suggested interpolating the adiabatic energy curves to improve the accuracy of the second derivatives, which we also do not use.^58^

An additional weakness of LZSH, and generally of algorithms based on PES’s curvature, stems from encountering discontinuities in electronic energies along trajectories. Such discontinuities are ubiquitous in multireference electronic structure methods and are caused by either orbital rotations into the active space or state flipping in the state-averaging procedure. While such discontinuities should be ideally avoided, general consensus tolerates “reasonable” discontinuities, providing that the character of the active electronic state has not changed. Such discontinuities then create spurious (and often very sharp) minima in *Z*_*ik*_. The LZSH algorithm spots such minima and, unable to recognize discontinuity from a true minimum, assigns hopping probabilities. Induced spurious hops then plague the dynamics. To mitigate the issue, we developed a simple scheme to detect spurious minima stemming from discontinuities in PESs. If LZSH finds a minimum in *Z*_*ik*_, it compares numerical second derivatives of *Z*_*ik*_ at the minimum (*Z̈*_*ik*,min_) and at the previous step (*Z̈*_*ik*,prev_). In the case of an avoided crossing, these second derivatives should vary smoothly. However, if a discontinuity is encountered, an abrupt change in the second derivative is expected. Therefore, we define a ratio α = |(*Z̈*_*ik*,min_ – *Z̈*_*ik*,prev_)/*Z̈*_*ik*,min_|. If α < 0.3, we consider PESs continuous. If α ∈ [0.3, 1.3], the code issues a warning to check the PESs manually. The numerical thresholds were set empirically. Note that if a sharp conical intersection is hit directly, values of α ≈ 1 are typically observed. If α > 1.3, the curvature change is too large – a typical feature of a discontinuity. The blocking algorithm is visually depicted in Figure 1. Note that more advanced schemes can also be developed, e.g., if one more step further is taken, yet this requires additional computational effort. A comparison between simulations performed with and without the discontinuity correction for the LZSH method is provided in the Supporting Information.

### 2.3 Fewest Switches Surface Hopping

Let us now move beyond the simple Landau–Zener model and introduce the fewest switches surface hopping approach. Originally proposed by Tully, FSSH is among the most widely used algorithms for describing transitions between electronic states.^16^ Its key objective is to minimize the total number of hops while ensuring that state populations remain consistent with the quantum mechanical probabilities, hence the term “fewest switches”. The hopping probability from state *i* to state *k* is given as

$$P_{i\to k}^{\mathrm{FS}}=\frac{2\Delta t}{\hbar |c_{i}{|}^{2}}\left(I[c_{k}c_{i}^{*}]H_{ki}-\hbar R[c_{k}c_{i}^{*}]\left\langle \phi_{k}|\frac{\partial \phi_{i}}{\partial t}\right\rangle \right)$$ 9

In the adiabatic basis, *H*_*ki*_ = 0 for *k* ≠ *i* and the first term in the above equation vanishes. The transition probability, therefore, simplifies to

$$P_{i\to k}^{\mathrm{FS}}=-\frac{2\Delta t}{|c_{i}{|}^{2}}R[c_{k}c_{i}^{*}]\left\langle \phi_{k}|\frac{\partial \phi_{i}}{\partial t}\right\rangle$$ 10

If this probability evaluates to a negative value, it is set to zero. Upon a successful hop from *i* to *k*, the nuclear coordinates continue to evolve classically on the *k*th adiabatic potential energy surface, and the velocity is rescaled (typically along the direction of the nonadiabatic coupling vector) to conserve the total energy. In certain instances, the system attempts a hop but lacks the necessary kinetic energy to transition to the new potential surface. These occurrences are known as “frustrated” hops. While there are various *ad hoc* approaches to handling such events, we follow Tully’s original method, where the hops are simply disregarded, since the simulations in this work are set up so that the particles always have enough kinetic energy to jump.

A known limitation of FSSH is that it does not inherently satisfy the internal consistency condition, implying that at any point in time, the fraction of trajectories on a given electronic state may not match the corresponding quantum probability. To address this issue, Granucci and Persico introduced an *ad hoc* decoherence correction that damps the electronic wave function coefficients, thereby restoring consistency.^18^ We adopt this correction in our simulations, and the specific parameters for decoherence will be detailed in subsequent sections.

### 2.4 Baeck–An Scheme, κTDC, and κFSSH

A key limitation of the FSSH algorithm is its need for TDC, which are typically computed from NACVs – an approach that can be too expensive, unstable, or even impossible for some electronic structure methods. While overlaps between electronic wave functions offer an alternative, this method requires careful phase tracking and is not supported in most standard quantum chemistry codes. To address this challenge, Baeck and An proposed an approximation for the magnitude of the NACV in the vicinity of the crossing point that relies solely on the curvature of the potential energy surface^48^

$$d_{ik}^{\mathrm{BA}}=\frac{1}{2}\sqrt{\frac{d^{2}Z_{ik}}{dR^{2}}\frac{1}{Z_{ik}}}$$ 11

where *Z*_*ik*_ = |*E*_*k*_ – *E*_*i*_|. We can use the chain rule and express the TDC according to Baeck and An, denoted as σ_*ik*_^λ^, as

$$\sigma_{ik}^{\lambda }=\frac{1}{2}\sqrt{\left(\frac{d^{2}Z_{ik}}{dt^{2}}-\frac{\ddot{R}}{\dot{R}}\frac{dZ_{ik}}{dt}\right)\frac{1}{Z_{ik}}}$$ 12

This expression is unsigned, due to the nature of the Baeck–An coupling. Should we now take the approximation that $\frac{dZ_{ik}}{dt}=0$ at the energy crossing (note that the Baeck–An coupling was derived for the energy crossing and its validity beyond it is only approximate^48^), we arrive at the commonly used expression for TDC^30^

$$\sigma_{ik}^{\kappa }=\frac{1}{2}\sqrt{\frac{d^{2}Z_{ik}}{dt^{2}}\frac{1}{Z_{ik}}}$$ 13

with the convention that for *k* > *i* the expression is positive, while for *k* < *i* one sets σ_*ik*_^κ^ = – σ_*ki*_^κ^. If the argument of the square root in eq 13 or eq 12 is negative, the element of the TDC is set to zero. The expression in eq 13 is often referred to as κTDC. A FSSH algorithm utilizing κTDC is subsequently referred to as κTSH in literature,^30^ however, we propose to call it rather κFSSH since it is the FSSH method just supplemented with κTDC. The expression in eq 12 is not commonly used and in this manuscript we will refer to it as λTDC. A FSSH algorithm utilizing λTDC will be analogously called λFSSH.

As mentioned in Section 2.2, hopping algorithms based solely on energies suffer from discontinuities in PESs. While spurious minima can be detected for LZSH and hops blocked, the situation is more complicated for both κ and λFSSH. κ/λTDC requires numerical second derivatives of *Z*_*ik*_ which are strongly affected by discontinuities (usually overestimating couplings). Since TDSE is propagated in κ/λFSSH using the κ/λTDC, the discontinuities will impact the electronic coefficients. However, it is not straightforward how to mitigate such problems since the hop can happen even long after encountering the discontinuity. Although we are aware of *ad hoc* corrections that attempt to address this issue,^51^ these solutions are typically system-specific and not generally applicable. We therefore do not include them in our simulations, consistent with ref 50, which also benchmarks the κFSSH method without any patches. As shown in the Supporting Information, the effect of the patch applied to LZSH is small and certainly does not account for the discrepancies between κFSSH and LZSH observed in azobenzene.

## 3 Computational Details

### 3.1 Analytic Potentials

We have used several analytical potentials to showcase the performance of the investigated TSH algorithms. The simplest and also the most popular two-state system one is the original simple avoided crossing (SAC) potential developed by Tully.^16^ The general diabatic potential, defined in atomic units, has the form

$$V_{00}(x)=-V_{11}(x)=A\mathrm{sgn}(x)(1-e^{-\mathrm{sgn}(x)Bx})$$ 14

$$V_{01}(x)=V_{10}(x)=Ce^{-x^{2}}$$ 15

where we have used *A* = 0.01 and *B* = −1.6. The parameter *C* will be specified at the corresponding places, as it is used to analyze the effect of the TDC width. To further challenge TSH algorithms, we present their performance on a system featuring three-state crossing. For direct comparison with Tully’s SAC model, we have expanded it by adding a zero-energy state between Tully’s original curves (see the first column of Figure 2). The resulting potential takes the form

$$V_{00}(x)=-V_{22}(x)=A\mathrm{sgn}(x)(1-e^{-\mathrm{sgn}(x)Bx}),\;V_{11}=0$$ 16

$$V_{01}(x)=V_{10}(x)=V_{12}(x)=V_{21}(x)=Ce^{-x^{2}},\;V_{02}=V_{20}=0$$ 17

where we have used *A* = 0.01 and *B* = −1.6. To ensure the corresponding three-state adiabatic potential has a zero-energy state, we set *V*_02_ = *V*_20_ = 0. Again, the coupling constant *C* will be specified at the appropriate places.

**Figure 2:**
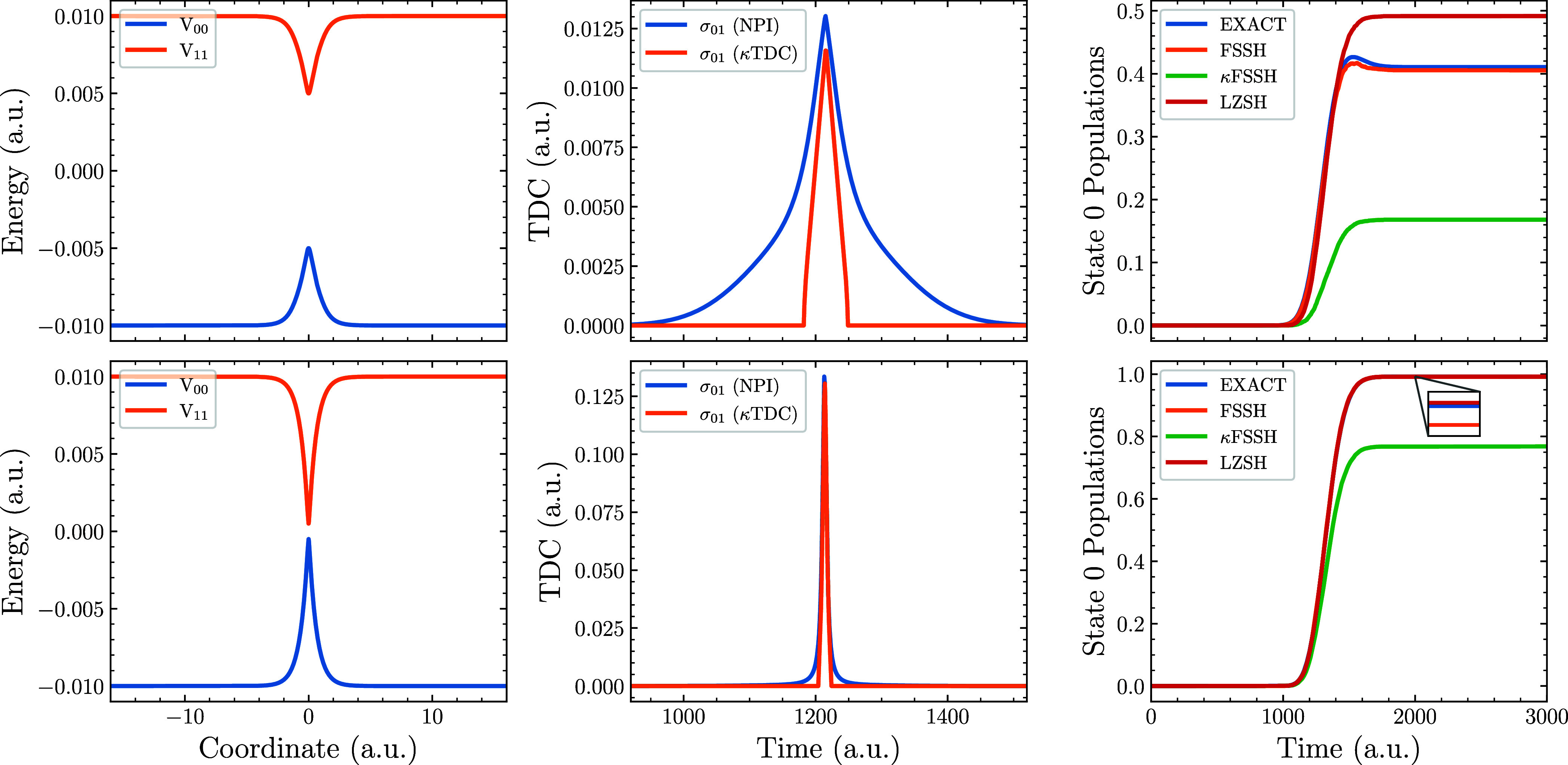
Nonadiabatic dynamics simulations on Tully’s Simple Avoided Crossing potential defined in ([Disp-formula eq14]) using two different sets of diabatic coupling constants for eq 15. The first row uses *C* = 0.005 a.u. while the second row uses *C* = 0.0005 a.u. The first column displays the potential energies in the adiabatic basis. The second column presents the TDC for a selected trajectory, while the third column shows the ground-state population throughout the simulation.

We also analyzed the SH algorithms on potentials with trivial crossings. The two-state model used for the analysis has the form

$$V_{00}(x)=0.0025(x+1{)}^{2},\;V_{11}(x)=0.0025(x-1{)}^{2}$$ 18

$$V_{01}(x)=V_{10}(x)=0$$ 19

For a three-state model with trivial crossings, we used

$$\begin{array}{c} V_{00}(x)=0.0025x^{2},\\ V_{11}(x)=A+0.0025(x-2{)}^{2},\\ V_{22}(x)=B+0.0025x^{2} \end{array}$$ 20

$$V_{01}=V_{10}=Ce^{-(x-2{)}^{2}},\;V_{12}=V_{21}=Ce^{-x^{2}},\;V_{02}=V_{20}=0$$ 21

where *A*, *B* and *C* are constants, which will be specified at the corresponding places.

### 3.2 Vibronic Coupling Model

The vibronic coupling (VC) model is an analytical Hamiltonian, typically formulated in the diabatic basis, that is fitted to describe the vibrational motion of a system.^59–61^ The model allows for highly efficient numerical evaluation of electronic structure quantities, even for highly dimensional systems. In this work, we employ a VC Hamiltonian representing an 8-dimensional model of the uracil cation. In the VC approach, the Hamiltonian is generally expressed as

$$H=H^{\left(0\right)}+W^{\left(1\right)}+W^{\left(2\right)}+W^{\left(3\right)}+\cdots$$ 22

where **H** ^(0)^ is the reference potential and the **W** terms represent the various vibronic coupling contributions.^60^ The Hamiltonian is typically written in terms of the mass-frequency scaled coordinate vector **Q**. We can write the reference potential as

$$H_{jj}(Q)=E_{j}+\underset{l}{\sum }V_{l}^{\left(j\right)}(Q)$$ 23

with *E*_*j*_ being the energy shift of an electronic state *j* and *V*_*l*_, depending on the anharmonicity of the mode *l*, being either harmonic or Morse potential written as

$$V_{l}^{\left(j\right)}=\frac{1}{2}\omega_{l}Q_{l}^{2}\vee V_{l}^{\left(j\right)}=d_{l}^{\left(j\right)}[e^{a_{l}^{\left(j\right)}(Q_{l}-Q_{l,0}^{\left(j\right)})}-1{]}^{2}+e_{l}^{\left(j\right)}$$ 24

where ω_*l*_ is the vibrational frequency of mode *l* and *d*_*l*_^(*j*)^, *a*_*l*_^(*j*)^, *Q*_*l*, 0_^(*j*)^, *e*_*l*_^(*j*)^ are constants specific for the vibrational mode *l* and electronic state *j*. The first-order coupling terms **W** ^(1)^ are given by

$$W_{jj}^{\left(1\right)}=\sum_{l}\kappa_{l}^{\left(j\right)}Q_{l}$$ 25

$$W_{jk}^{\left(1\right)}=\sum_{l}\lambda_{l}^{\left(jk\right)}Q_{l}$$ 26

where κ_*l*_^(*j*)^ is a constant for the vibrational mode *l* in an electronic state *j* and λ_*l*_^(*jk*)^ denoting a coupling constant between electronic states *j* and *k* for a vibrational mode *l*. If the Hamiltonian in eq 22 is truncated after the first-order terms, the model is referred to as the linear vibronic coupling (LVC) model.^62^ In this work, we also consider diagonal elements of **W** ^(2)^ and **W** ^(4)^ in the form of

$$W_{jj}^{\left(2\right)}=\frac{1}{2}\sum_{l}\gamma_{l}^{\left(j\right)}Q_{l}^{2}$$ 27

$$W_{jj}^{\left(4\right)}=\frac{1}{24}\sum_{l}k_{l}^{\left(j\right)}Q_{l}^{2}$$ 28

where γ_*l*_ and μ_*l*_ are constants. All modes, constants, and reference potentials used in the VC models in this work are provided in the Supporting Information.

### 3.3 Nonadiabatic Dynamics on Analytic Potentials

We performed numerically exact nonadiabatic dynamics by solving TDSE in the diabatic basis using the split-operator method.^63,64^ In all simulations, the initial wave function was chosen as a Gaussian wavepacket in a form

$$\Psi_{0}(x,t)=\Omega \exp(-(x-x_{0}{)}^{2}+ip_{0}(x-x_{0}))$$ 29

where *x*_0_ and *p*_0_ represent the initial position and momentum, respectively, *i* is the imaginary unit and Ω is the normalization factor. Simulations were carried out on a spatial domain of [−24 a.u., 48 a.u.] (or [−10 a.u., 10 a.u.] for trivial crossings) discretized into 8192 grid points, using a mass of 2000 a.u. and a time step of 10 a.u. For Tully’s potentials (eqs 14 and 16) we have set the initial momentum to 15 a.u., all other simulations on model potentials used zero initial momentum.

To carry out FSSH simulations on analytic potentials, we employed the norm preserving interpolation (NPI) scheme to approximate the TDC.^65,66^ Adiabatic surface gradients were obtained using first-order finite differences with a step size of 0.001 a.u. The time step used was set to 1 a.u. and each simulation on analytic potential used 10,000 trajectories.

### 3.4 Nonadiabatic Molecular Dynamics on *Ab Initio* Potentials

Three molecular systems are investigated with *ab initio* nonadiabatic dynamical simulations in this work: photoisomerization of S_1_ excited *cis*-stilbene and *trans*-azobenzene, and photodissociation of S_2_ excited cyclobutanone. For *cis*-stilbene. We employed the multireference configuration interaction with singles and doubles (MRCISD) method based on the semiempirical OM3 Hamiltonian and state-averaged complete active space self-consistent field (SA-CASSCF) approach. The SA-CASSCF calculations utilized an active space comprising two electrons in two π and π* orbitals, denoted as (2,2), reproduced from a previous study.^57^ In contrast, the OM3-MRCISD method, due to its computational efficiency, enabled the use of a significantly larger active space, such as (12,17), also reported in earlier work.^39^ All cis-stilbene trajectories were initiated in the S_1_ state (considering only the lowest two states), with initial geometries and momenta sampled from a harmonic Wigner distribution around the MP2/6-31G* minimum of *cis*-stilbene, assuming a temperature of 298.15 K, see ref 38, for more details. The SA-CASSCF calculations were carried out using the BAGEL package,^67^ while OM3-MRCISD computations were performed with the MNDO v7.0 code.^68^ All dynamical simulations were executed within our molecular dynamics code ABIN and the time step was set to 5 a.u.^69^

For the *trans*-azobenzene, we have performed only the OM3-MRCISD calculations, also with the active space of (12,17).^39^ All the trajectories start in the S_1_ state (considering only the lowest two states) and the initial conditions are sampled using a harmonic Wigner distribution around the B3LYP/6-31G* minimum of *trans*-azobenzene, also assuming a temperature of 298.15 K.

The cyclobutanone simulations with extended multistate complete active space second-order perturbation theory (XMS-CASPT2) were done as described in ref 37. For the CASSCF simulations, we employed the same active space, initial conditions, and general strategy as in ref 37, but we did not restart the trajectories in the ground state.

Each OM3-MRCISD simulation was performed using 500 trajectories. The number of failed trajectories for these simulations did not exceed 20, and all of them were due to poor energy conservation. The cis-stilbene SA-CASSCF simulations employed 168 FSSH trajectories, 89 κTSH trajectories, and 91 LZSH trajectories. All cyclobutanone simulations comprised 119 trajectories.

### 3.5 Trajectory Analysis

The principal quantities of interest in these simulations are the electronic state populations. At any given time *t*, the population of a specific state is computed by taking the fraction of trajectories in that state relative to the total number of trajectories still active. In the event that a simulation terminates immediately after a jump to the ground state, we assume that the trajectory remains in the ground state for the rest of the simulation. Consequently, this trajectory continues to contribute to the ground-state population even after the simulation has failed.

Because each population can be viewed as a realization of a multinomial random variable at each time point, we estimate its confidence interval by invoking the normal approximation. Specifically, for the *k*th state, the confidence interval at time *t* is given by

$$\Delta p_{k}(t)=\pm z\sqrt{\frac{p_{k}(t)(1-p_{k}(t))}{N(t)}}$$ 30

where *p*_*k*_(*t*) denotes the population of state *k* at time *t*, *N*(*t*) is the number of trajectories still considered at the time *t*, and *z* is the 97.5% quantile of the standard normal distribution (i.e., *z* ≈ 1.96) for a two-sided 95% confidence interval.

## 4 Results

We begin by applying the LZSH, FSSH, and κFSSH algorithms to various analytic potentials, evaluating their theoretical performance against the numerically exact solution of the time-dependent Schrödinger equation. Next, we extend our analysis to more realistic systems, examining how the curvature-based algorithms perform on the LVC model of Uracil. Finally, we shift to real molecular systems which feature less forgiving PESs for the curvature-based algorithms. Finally, the comparison of these algorithms in *ab initio* simulations of stilbene, azobenzene, and cyclobutanone will be provided.

### 4.1 Model Potentials

#### 4.1.1 Two-State Avoided Crossing Model

Let us start by examining how the various algorithms perform on a two-level system, specifically Tully’s SAC potential (see Section 3.1). The results for this two-level case with diabatic coupling constant *C* = 0.005 a.u. are shown in the top row of Figure 2. In the bottom row of the same figure, we use the same potential expression but with coupling constant *C* = 0.0005 a.u. This allows us to assess how sensitive the final electronic populations are to the exact shape of the TDC.

Inspecting the TDCs from the top row of Figure 2, it is clear that NPI TDC is wider than the κTDC. This discrepancy directly affects the ground-state populations (right panels) and κFSSH performs poorly compared to LZSH and especially FSSH. To confirm that the culprit is indeed the TDC approximation, we repeated the simulations with a modified diabatic coupling which causes the TDC to be more time localized. This improves the agreement between κTDC and NPI TDC simulations, shown also in ground-state populations although one still observes that the LZSH algorithm outperforms the κFSSH method. Note that λFSSH fully correlates with κFSSH. Hence, LZSH appears to be a more robust choice in this particular two-state problem. In both cases, FSSH with NPI TDC aligns with the exact results.

#### 4.1.2 Three-State Avoided Crossing Model

Next, we push the algorithms further by considering a three-state analogue of Tully’s SAC potential, defined in eqs 16 and 17 with *C* = 0.003 a.u. The corresponding results are shown in Figure 3. As with the two-level model, we also present (in the bottom row) results obtained by using a modified coupling with *C* = 0.0003 a.u. Once again, κTDC is too narrow compared to NPI TDC. However, we now encounter additional problems stemming from the three-state nature of the model. While the NPI method correctly predicts TDC to be zero between states 0 and 2, the κ approach assigns nonzero TDC elements to every pair of states.

**Figure 3:**
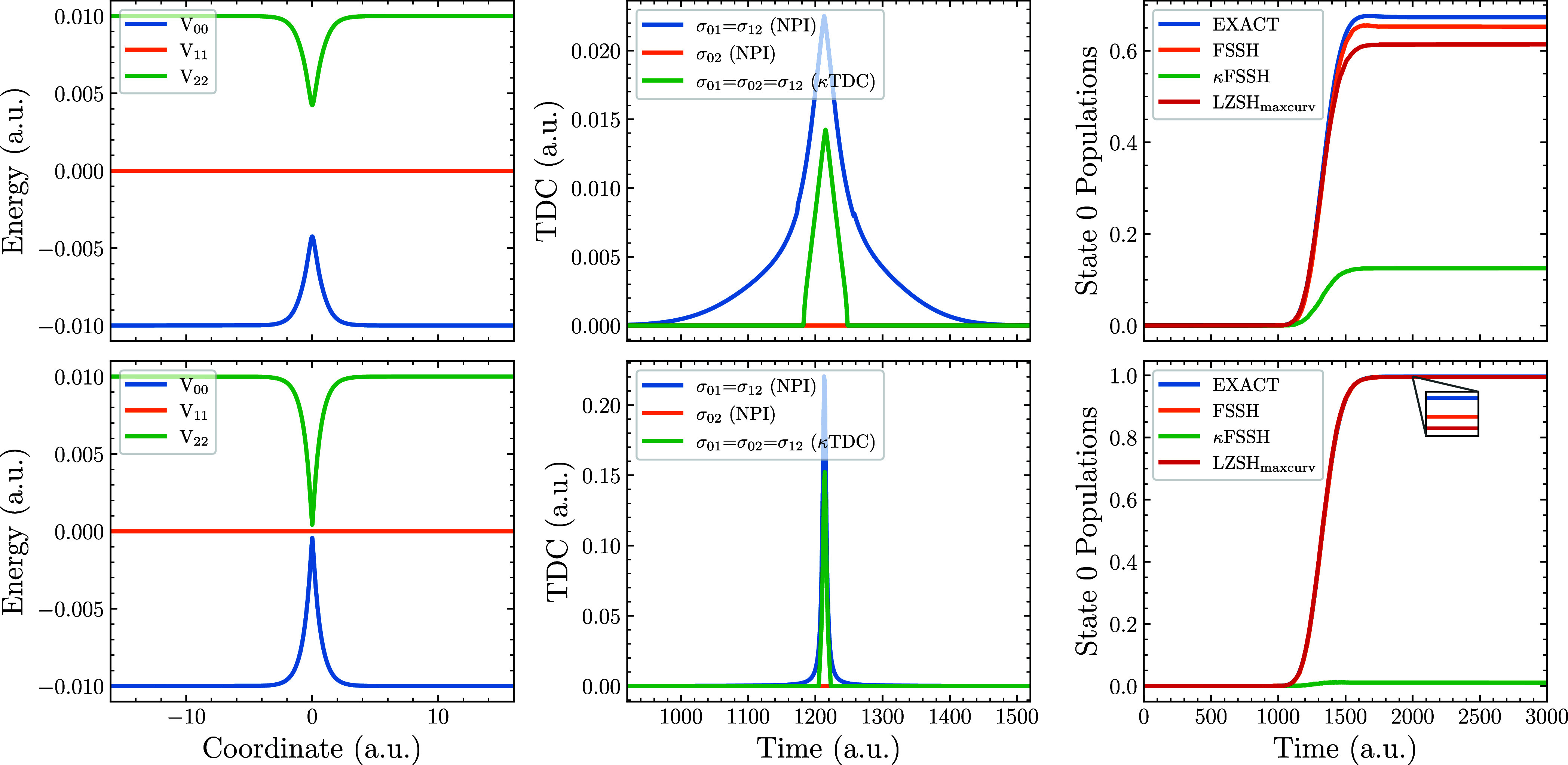
Nonadiabatic dynamics simulations on a three-state crossing potential defined in ([Disp-formula eq16]) using two different sets of diabatic coupling constants for eq 17. First row uses *C* = 0.003 a.u. while the second row uses *C* = 0.0003 a.u. The first column displays potential energy curves in the adiabatic basis. The second column presents the TDC for a selected trajectory, while the third column shows the ground-state population over the course of the simulation.

A closer inspection of the underlying potential clarifies these failures. Analytically, one can show that for the chosen three-state model, the NACV between states 0 and 2 is vanishing. The NPI method captures this fact; the κTDC approach, based solely on adiabatic curvature, does not. One can further demonstrate that $\frac{{\ddot{Z}}_{01}}{Z_{01}}=\frac{{\ddot{Z}}_{02}}{Z_{02}}=\frac{{\ddot{Z}}_{12}}{Z_{12}}$ . Under these conditions, the κTDC is the same for all pairs of states. We should also note that the κTDC underestimates the magnitude of TDC, which will become a problem in the following analysis. Finally, we add that λTDC and κTDC again exhibited the same shape.

The results also highlight an excellent performance of LZSH in its maximum second derivative version (LZSH_maxcurv_), contrasting with the poor performance of κFSSH. The success of LZSH_maxcurv_ dwells in its ability to recognize the interacting states by maximum curvature of the energy difference. The nearest state version introduced in Section 2.2 performs poorly in this case since it transfers population solely to state 1 and neglects state 2 completely. Similarly, we have tested a version where the hop goes to a state with maximum hopping probability, leading again to the population of only state 1.

In both cases, FSSH with NPI TDC is again closest to the exact results. Hence, if analytic couplings are available, FSSH is the best approach from those selected. However, if couplings are not available, it appears beneficial to prioritize LZSH_maxcurv_ over κFSSH.

#### 4.1.3 Trivial Crossing Models

The varying TDC magnitude across different methods naturally raises the question of how surface hopping algorithms perform when applied to potential energy surfaces exhibiting trivial crossings. In such cases, the associated nonadiabatic coupling becomes singular at the crossing point.

Figure 4 presents the nonadiabatic dynamics computed for the two-state model defined in eq 18 and three-state potential defined in eq 20, using three different sets of parameters.

**Figure 4:**
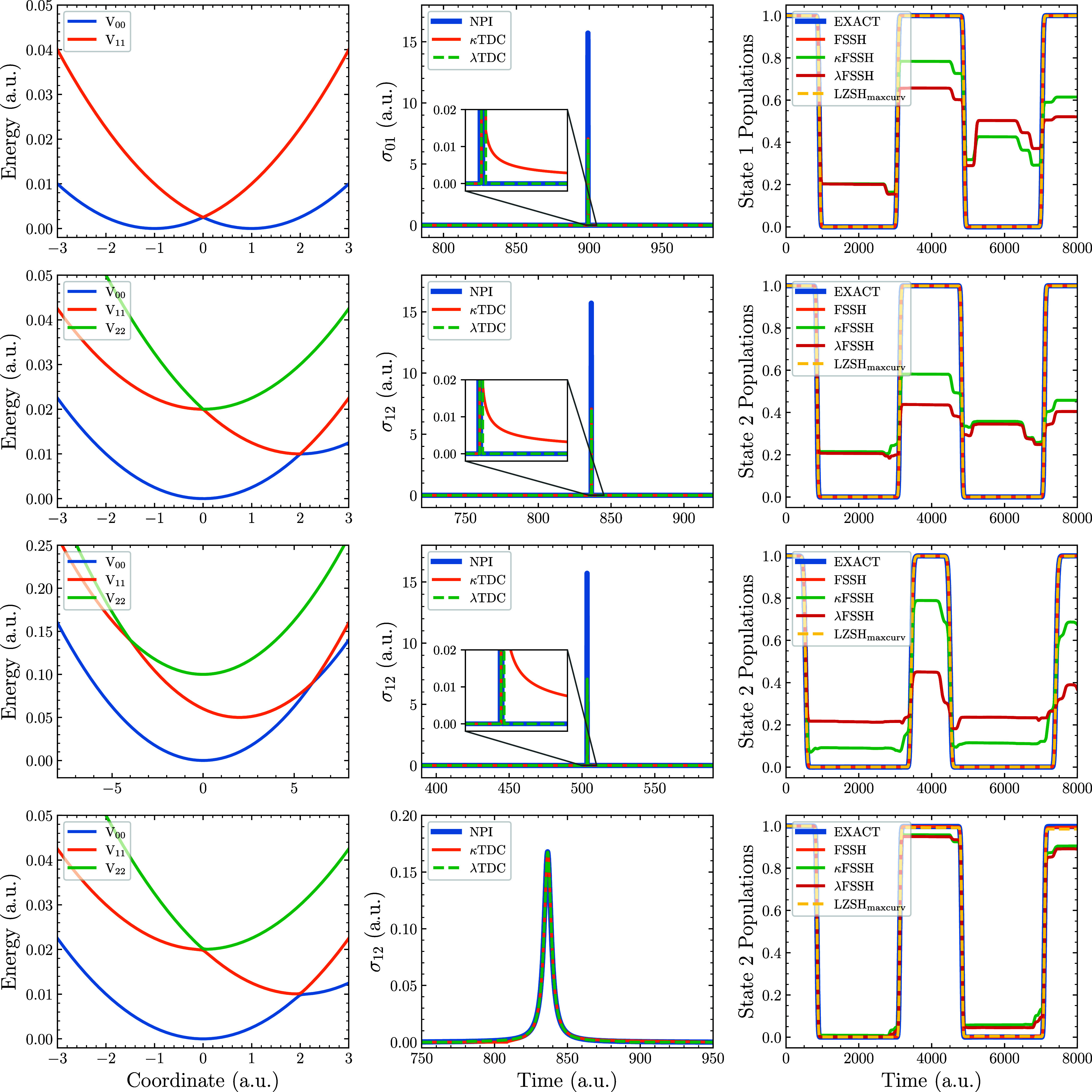
Nonadiabatic dynamics computed on the model potential of eq 18 (top row) and eq 20 (remaining rows). The left column depicts the adiabatic potential energy curves, the middle column is the TDC of a random trajectory near the first crossing, and the right column displays the corresponding time-dependent adiabatic state populations. In the second row, the diabatic potential parameters are set to *A* = 0.01 a.u., *B* = 0.02 a.u. and *C* = 0 a.u. The third row uses *A* = 0.05 a.u., *B* = 0.1 a.u. and *C* = 0 a.u., increasing the energy separation between states. The last row introduces a small Gaussian coupling with *A* = 0.01 a.u., *B* = 0.02 a.u. and *C* = 2 × 10^–4^ a.u., which perturbs the crossing topology while maintaining full deexcitation of the initially populated state. All simulations started at the highest adiabatic state at the point *x* = −7 a.u.

The first row of Figure 4 corresponds to the potential defined in eq 18, which exhibits a TDC singularity at the point *x* = 0. As we see, the magnitude of the NPI TDC is sufficient to allow FSSH a 100% transfer to the ground state. The LZSH_maxcurv_ algorithm also predicts a full transfer to the ground state due to the infinitely small gap between the states at the crossing. However, as we have already seen in the Figures 2 and 3, κFSSH and λFSSH underestimate the magnitude of the TDC and the size of the coupling is simply not high enough to predict the full population transfer. Due to the insufficient magnitude of the TDC in these methods, the electronic coefficients also do not reach the full transfer at the first crossing, making the subsequent jumps even further from the exact solution. We should also note that the κTDC has a small tail due to the $\frac{dZ_{jk}}{dt}=0$ approximation, contrary to the λTDC. This small tail partially compensates for the insufficient peak magnitude of the coupling and enhances the performance over λTDC. However, this is only a result of the compensation of errors.

In the second row of Figure 4, the potential energy surfaces exhibit two trivial crossings. The NPI TDC and LZSH_maxcurv_ algorithm, again, reproduce the population transfer perfectly. Both κFSSH and λFSSH approaches perform poorly in this scenario, owing mainly to the size of the coupling, leaving residual population on S_2_ rather than effecting a full transition. We should also note here that κTDC and λTDC predict nonzero coupling between S_0_ and S_2_ at the first crossing that is not high enough to allow for a population transfer to S_0_ but affects the overall electron dynamics.

In the third row of Figure 4, the same diabatic potential as in the second row is used, but the energy gap between adjacent surfaces has been increased by a factor of 5, which shifts the first crossing point away from the S_2_ minimum. Nonetheless, since $\frac{d^{2}Z_{ik}}{dt^{2}}$ is not necessarily zero away from the crossing, the κTDC has a nonzero tail which is, in this case, not negligible. Due to this tail, we see more accurate population transfer for κFSSH than λFSSH. Still, the performance of FSSH and LZSH_maxcurv_ remains unchallenged. Histogram of the hopping positions around the first crossing for this model can be found in the Supporting Information.

In the bottom row of Figure 4, we introduce a localized Gaussian diabatic coupling with a peak magnitude of 10^–4^ a.u. This modification perturbs the energy crossing only slightly while preserving the full transfer at the crossing points. FSSH with NPI TDC provides, as expected, exact populations. The LZSH_maxcurv_ algorithm also yields quantitatively correct populations. Moreover, the Gaussian coupling renders the κTDC/λTDC profile more similar to the NPI TDC, such that κFSSH/λFSSH results become acceptable. We should still note a nonzero κTDC and λTDC between the S_0_ and S_2_ state at the crossings, which causes small deviations of populations using these methods.

Note that for all the three-state models in Figure 4, both LZSH_maxcurv_ and LZSH_nearest_ perform equally since the interacting state with the largest *Z̈*_*ik*_ is always the nearest state. The difference in the algorithms appears only when interaction over multiple states occurs, which is not a common case in photodynamics.

In summary, Figures 2 and 3 illustrate the importance of obtaining a reliable TDC. The limited spatial width inherent to κTDC does introduce errors, yet the more serious failures of the κTDC and λTDC schemes arise from structural flaws that assign nonadiabatic coupling even between electronic states that should not be coupled. Figure 4 further demonstrates that, in the case of trivial crossings, the κFSSH and λFSSH methods inherently fail to predict correct population transfer, whereas the LZSH_maxcurv_ algorithm remains robust and therefore constitutes a superior choice for such systems.

Finally, we note that there is currently an initiative to apply κFSSH also for extended system^70^ which, however, often exhibit trivial crossings. Vogt et al. compare FSSH results using κTDC (coined TDBA) as well as standard TDC in ref 70, showing notable discrepancies for κTDC. The authors suggest caution when using κTDC as its two state nature leads to population leakage to other states. In light of our results, we would advocate for using LZSH which does not suffer from such issues as is also seen in other works.^58^

### 4.2 Uracil Cation VC Model

Next, we consider a more realistic and complex system to evaluate the performance of surface hopping algorithms: the vibronic coupling model for the uracil cation, which exhibits a three-state conical intersection. The curvature-based methods are expected to fail in such a case. As a reference, we utilize multiconfigurational time-dependent Hartree (MCTDH) dynamics data reported in ref 71, based on an eight-mode vibronic coupling model developed in ref 72 and further described in Section 3.2.

The MCTDH simulations were performed in the diabatic representation, with the initial wavepacket having projections of 94% onto the adiabatic state D_2_, 5% onto D_1_, and 1% onto D_0_. Our SH simulations were initialized to reflect these population distributions. A comparative summary of the population dynamics obtained with the different SH algorithms is presented in Figure 5.

**Figure 5:**
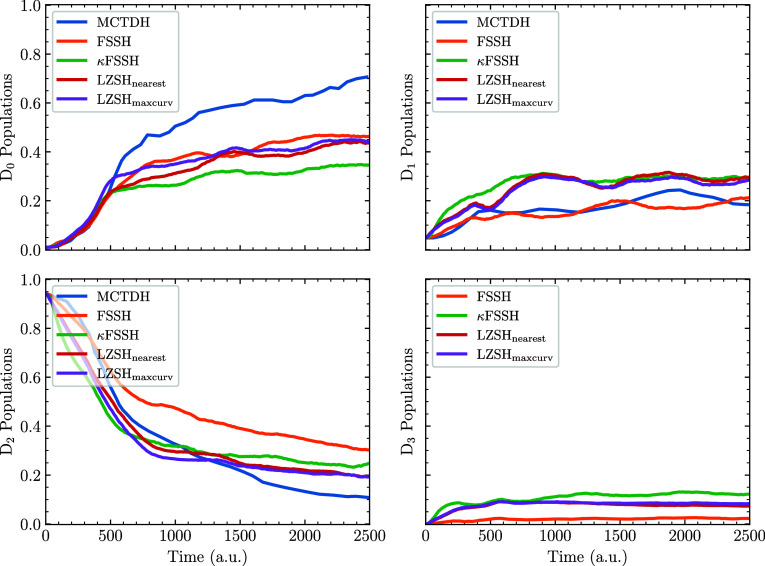
Population dynamics of the uracil cation computed using an eight-mode vibronic coupling model. Initial conditions were sampled from a Wigner distribution. Trajectories were propagated with a time step of 10 au for 250 iterations, following the protocol outlined in ref 71. The initial electronic state was a mixed state composed of 94% D_2_, 5% D_1_, and 1% D_0_ populations.

As shown in Figure 5, none of the tested surface hopping algorithms quantitatively reproduce the MCTDH population dynamics, in agreement with prior studies.^71^ We note here that the exact factorization-based surface hopping (SHXF) method is in better agreement with MCTDH as shown in ref 71. Our focus here is on the qualitative differences between the SH approaches. Among the methods analyzed, the FSSH algorithm exhibits noticeably distinct population dynamics. FSSH correctly predicts negligible population transfer to the D_3_ state, in agreement with previous *ab initio* studies.^73^

In contrast, curvature-driven approaches inaccurately predict nonzero population transfer to D_3_, indicating a limitation in capturing the correct coupling mechanism for this state. Furthermore, the κFSSH method underestimates the population transfer to D_0_ and overestimates the transfer to D_1_, suggesting slightly inferior performance compared to the LZSH algorithms.

Interestingly, the LZSH_maxcurv_ and LZSH_nearest_ variants yield nearly indistinguishable results, implying that for complex systems such as this, the specific choice of the three-state LZSH variant may not significantly affect the overall population dynamics.

In general, all methods qualitatively capture the uracil dynamics. Although discrepancies between the methods can be seen, none of them can be claimed superior, and all seem suitable for simulations of molecular systems. The uracil example just demonstrates how multidimensional models can be forgiving to nonadiabatic dynamics methods compared to one-dimensional models. However, the presented VC model still lacks one feature of real molecular systems: discontinuities in PESs appearing upon orbital rotations into the active space. Thus, a true benchmark on real molecular systems is essential.

### 4.3 Photoisomerization of *cis*-Stilbene and *trans*-Azobenzene

To assess the performance of trajectory surface hopping algorithms on molecular systems, we first selected the photoisomerization processes of *cis*-stilbene and *trans*-azobenzene, both of which have been extensively studied in previous theoretical simulations.^38,74–78^ We have selected these molecules, since a small variation of the electronic structure method leads to large variations in the outcome of the photodynamics. Furthermore, we use them as an example of systems with highly fluctuating total energy, due to the orbital rotation issues. Analytical model potentials presented above show that κFSSH algorithms fail to reliably capture the dynamics of three-state intersections or trivial crossings. These features, however, are rare in molecular systems and do not occur in either *cis*-stilbene nor *trans*-azobenzene. Thus, the κFSSH or LZSH simulations for both molecules should not suffer from the corresponding approximations of κTDC.

Nevertheless, it is important to note that both LZSH and κFSSH methods rely on potential energy derivatives, and any discontinuities in potential energy surfaces can introduce numerical instabilities. A small energy discontinuity is not unusual in photodynamics, especially when active space-based methods are used. While our LZSH algorithm is armored with our empirical shield to detect discontinuity-induced hops, κFSSH is left naked due to its nature (see discussion in Sections 2.2 and 2.4). The electronic-state populations and photoproduct populations of *cis*-stilbene and *trans*-azobenzene as a function of time are summarized in Figure 6.

**Figure 6:**
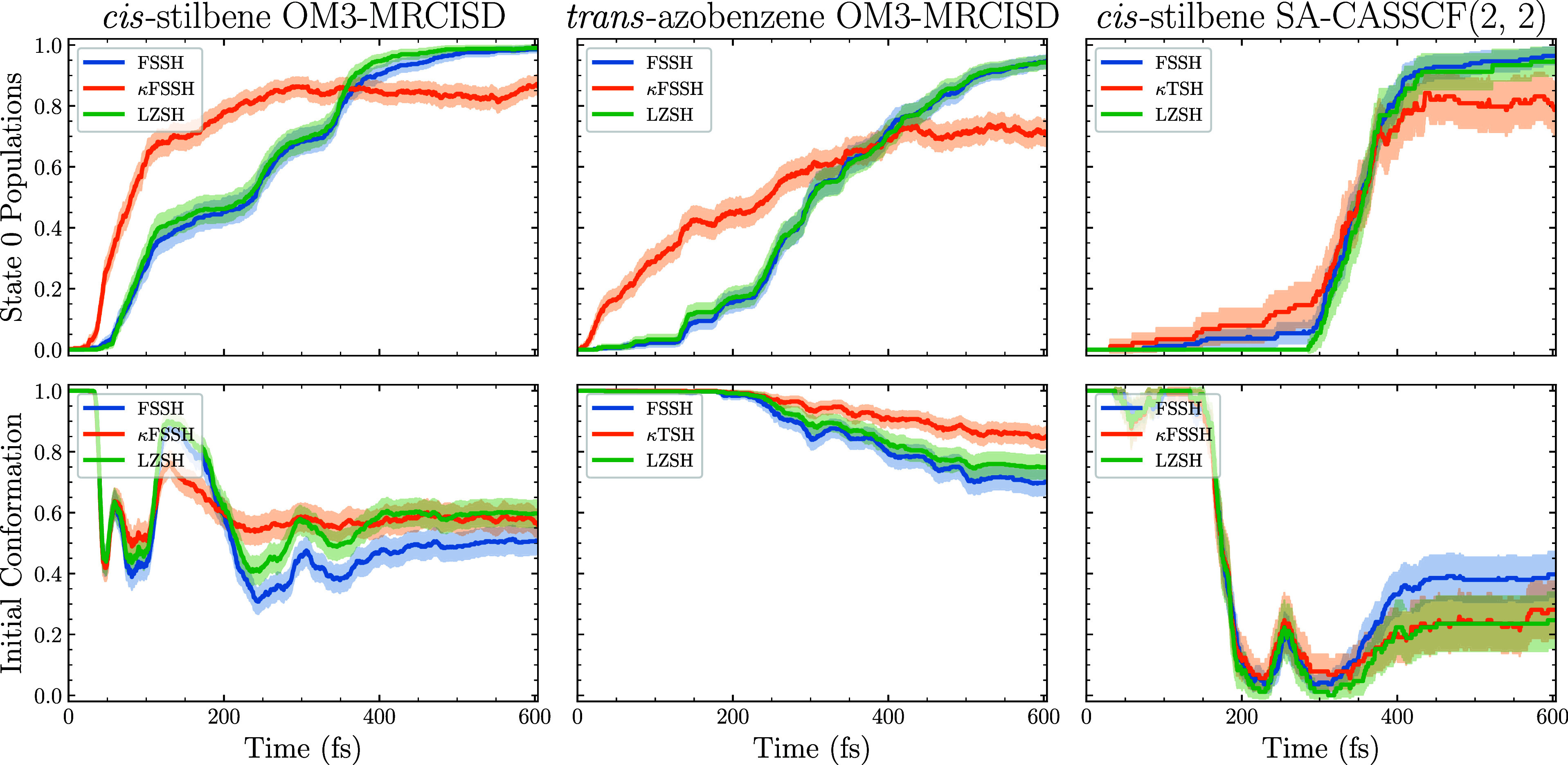
*Ab initio* molecular dynamics simulations of *cis*-stilbene and *trans*-azobenzene using FSSH, κFSSH, and LZSH. Since only two electronic states were included, the original (two-state) formulation of LZSH was applied. The top row displays the electronic state populations over time, while the bottom row shows the populations of the initial conformations as a function of time.

The first two columns present the OM3-MRCISD dynamics for *cis*-stilbene and *trans*-azobenzene. As it is clear from the results, the κFSSH algorithm fails to correctly describe the electronic populations while FSSH and LZSH yield nearly identical outcomes. This failure of κFSSH is not surprising, as OM3-MRCISD simulations are known to suffer from discontinuities in potential energy surfaces and lack proper energy conservation.^38^ Consequently, numerical derivatives, crucial for the κFSSH algorithm, are ill-defined at these discontinuities. Although LZSH also suffers from discontinuities, it can cope with them better, partially thanks to the algorithm form blocking discontinuity-induced hops described in Section 2.2.

To test this hypothesis, we performed analogous simulations at the cc-pVDZ/SA-CASSCF­(2,2) level of theory, where potential energy surfaces are smooth and almost free of the aforementioned discontinuity issues.^38^ As shown, the agreement between κFSSH and the other algorithms improves significantly on the CASSCF potential energy surface. However, even in these simulations, the LZSH method remains in closer agreement with FSSH, and we thus observe no clear advantage of using κFSSH over LZSH.

### 4.4 Photodissociation of Cyclobutanone

As a final molecular model, we chose the cyclobutanone molecule that has been recently studied extensively within the photodynamics prediction challenge.^79^ We focus on the *S*_2_ state of Rydberg character. Cyclobutanone was selected not only because it is currently the most studied molecule in photodynamics but also because the lowest three states all interact at the same time, as reported in ref 37. We compare the dynamics on both the XMS-CASPT2 and CASSCF potentials, see Figure 7.

**Figure 7:**
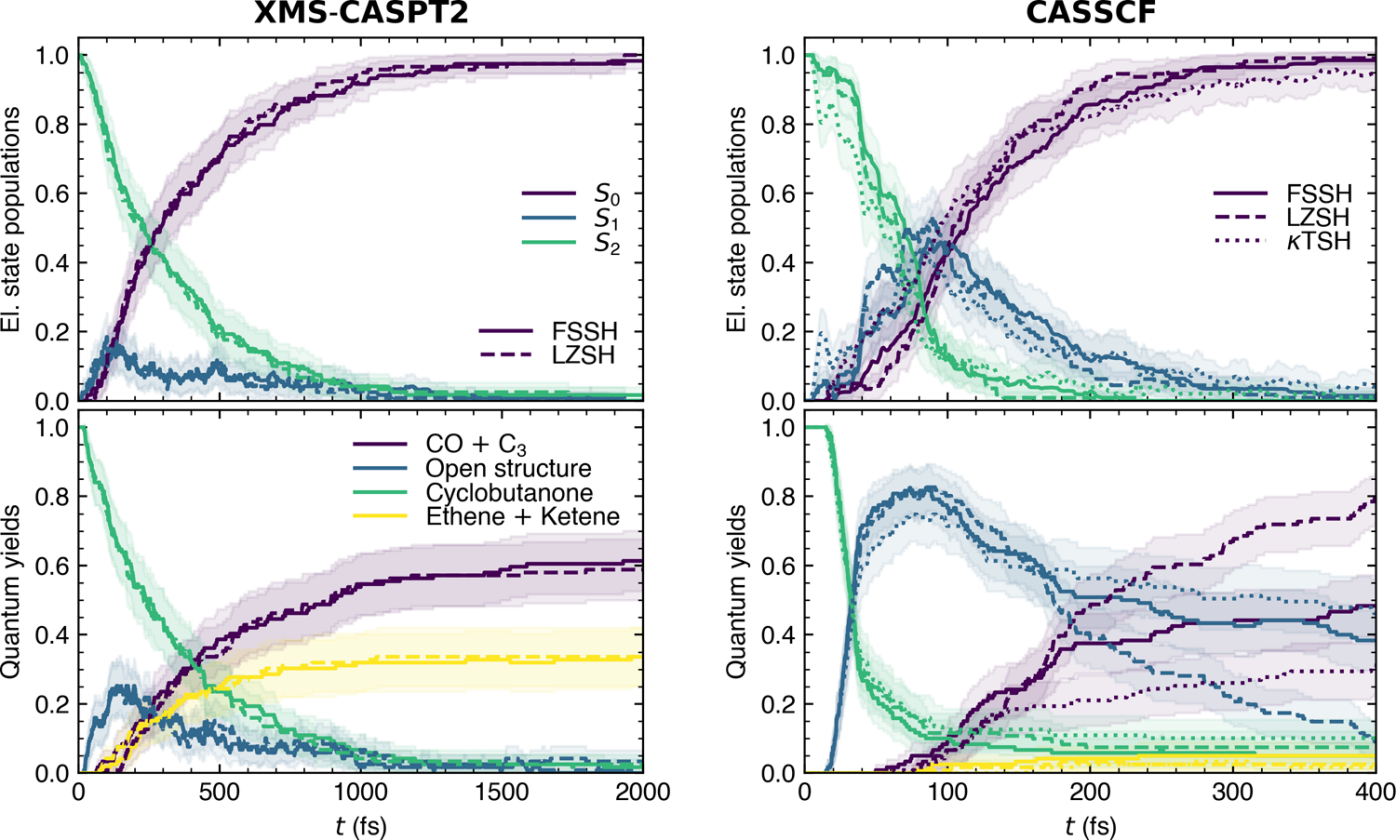
Nonadiabatic dynamics simulations of cyclobutanone upon excitation to S_2_ based on XMS-CASPT2­(8,8)/aug-cc-pVDZ (left column) and SA2-CASSCF­(8,8)/aug-cc-pVDZ. Initial conditions and other details are available in ref 37.

Let us start with the XMS-CASPT2 simulations where we compare our trajectories from the prediction challenge^37^ with a new set of LZSH trajectories using the nearest-state algorithm (LZSH_nearest_ was shown to be equal to LZSH_maxcurv_ at the uracil cation model). The results show an exceptional agreement in both electronic populations and time-dependent quantum yields demonstrating a negligible effect of the nonadiabatic algorithm on cyclobutanone dynamics. LZSH_nearest_ performs well despite the three-state character of the open-ring conical intersection.^37^ Such a result highlights the capability of LZSH to deal with multistate problems.

Focusing on the CASSCF dynamics, including also κFSSH, we observe larger variations of the data compared to the XMS-CASPT2 results. Focusing on electronic populations, the results for all methods are still within error bars most of the time, yet they do not overlap as nicely as XMS-CASPT2 populations. The most notable difference can be seen at the very beginning where κFSSH predicts a fast drop of the S_2_ population to 0.8, yet it is soon compensated. Discrepancies appear when scrutinizing the time-dependent populations of the reaction products. While all methods predict the same decay of the cyclobutanone structure and negligible quantum yield of ethene and ketene, they disagree on the intermediate open structure and CO + C_3_ yields. Note that part of this discrepancy can be attributed to instabilities in CASSCF which cause up to 20 trajectories to fail out of 121, with LZSH being the most stable. All the data are summarized in Table 1. Overall, all employed methods (FSSH, LZSH and κFSSH) perform well.

**Table 1:** Summary of Excited-State Lifetimes and Quantum Yields at the End of the Simulations

		lifetime (fs)		quantum yield		
el. str.	method	τ_S_2__	τ_S_1__	CO + C_3_	ethene + keten	open str.
CASSCF	FSSH	73.1 ± 2.0	59.4 ± 2.6	0.48 ± 0.09	0.05 ± 0.04	0.38 ± 0.09
	LZSH	68.0 ± 1.9	56.8 ± 2.6	0.79 ± 0.07	0.03 ± 0.03	0.09 ± 0.05
	κFSSH	63.3 ± 2.3	57.0 ± 2.6	0.31 ± 0.08	0.02 ± 0.02	0.46 ± 0.09
CASPT2	FSSH	334 ± 12	66 ± 10	0.61 ± 0.09	0.34 ± 0.08	0.00 ± 0.00
	LZSH	333 ± 13	64 ± 10	0.59 ± 0.09	0.33 ± 0.08	0.03 ± 0.03

Finally, let us comment on the algorithm for blocking discontinuity-induced hops in LZSH. The algorithm blocked 65 hops with an average hopping probability of 0.39 for CASSCF and 76 hops with an average probability of 0.15 for XMS-CASPT2. Hence, the algorithm blocked the equivalent of 25 hops for CASSCF and 11 hops for XMS-CASPT2. While not critically large, these hops would affect the dynamics notably.

## 5 Conclusions

In this work, we critically assess the performance of FSSH, κFSSH, λFSSH and LZSH methods across a diverse set of benchmark models, ranging from analytic two- and three-state avoided crossings to trivial crossings and realistic molecular applications including the uracil vibronic-coupling model, *cis*-stilbene, *trans*-azobenzene, and cyclobutanone. To be capable of such comparison, we first had to address one of the fallbacks of LZSH – its two-state nature. Hence, we developed two generalized multistate variants of LZSH: the maximum curvature version (LZSH_maxcurv_) and the nearest state version (LZSH_nearest_). Both variants perform equally and successfully reproduce FSSH population dynamics for all molecular test cases, with the exception of the three-state avoided crossing model where LZSH_maxcurv_ outperforms LZSH_nearest_. Thus, we further use the term LZSH without distinguishing its two new flavors.

Comparing the methods across the whole benchmark set, all curvature-based methods (κFSSH, λFSSH and LZSH) exhibit systematic deviations from both numerically exact dynamics and benchmark FSSH results under a variety of test conditions. Finally, the results clearly demonstrate that both κFSSH and λFSSH rarely outperform LZSH, even in low-dimensional test systems.

We can identify two main scenarios where κFSSH and λFSSH underperform compared to LZSH and FSSH. First, the curvature driven methods suffer from discontinuities common for methods like OMx-MRCISD or CASSCF. LZSH appears to be less prone to discontinuity-induced hopes and, hence, more robust in this sense. However, all mentioned methods perform adequately for well-behaved PESs. Second, trivial crossings involving multiple states constitute a major challenge, since both κFSSH and λFSSH tend to leak population into states that do not interact. Again, both multistate variants of LZSH are not prone to such behavior. While quite rare in gas-phase photochemistry, trivial crossings in the manifold of states are typical in solid phase. A recent study expanding the TSH technology to extended systems explored the behavior of κFSSH (coined FSSH with Baeck–An coupling) in such a scenario.^70^ The authors observe a strong leakage of population in κFSSH and attribute it to the two-state nature of κFSSH, in line with our observation. Following this discussion, we would advocate for applying LZSH in extended systems and exploring its quality as it appears to be a suitable candidate.

Apart from the two-state assumption and discontinuities, further limitations of κFSSH/λFSSH appear to stem from the shape of the coupling. In particular, the curvature-driven approximated TDCs tend to underestimate peak values and mischaracterize their spatial profiles. In multistate intersections, they misallocate coupling strength across electronic states, resulting in unphysical transitions. Moreover, any noise or discontinuity in the potential energy surface has an amplified effect due to the dependence on second-order temporal derivatives, undermining the reliability of curvature-based TDCs.

All curvature-based TSH methods (including LZSH) suffer from discontinuities and require additional safeguards to function correctly. In this work, we introduced a blocking algorithm to prevent discontinuity-induced hops in LZSH. This correction enhances the algorithm’s stability on discontinuous potential energy surfaces; however, its impact in our simulations is limited, as demonstrated in the Supporting Information. Generally, LZSH shows lower sensitivity to artifact of PESs such as discontinuities compared to κFSSH/λFSSH. Any deployment of κFSSH/λFSSH should include similar protective strategies if reliable dynamics are to be obtained.

Although FSSH most faithfully reproduces electronic populations when NACVs can be computed, LZSH retains certain conceptual advantages, such as the correct phase treatment, independence of decoherence corrections or a more transparent physical grounding in Landau–Zener theory. The assumptions under LZSH are also suitable for such a topologically delicate feature as trivial crossings. Furthermore, lack of NACVs offers a significant speed-up of simulations compared to FSSH (this is also a merit of κFSSH). These features and its robustness suggest that LZSH is not merely a fallback method, but a compelling choice for nonadiabatic dynamics, particularly in combination with on-the-fly electronic structure methods.

Future developments could proceed along several axes. First, LZSH and κFSSH could potentially be combined in simulations where disagreement between the algorithms would hint at possible issues with the multistate assumption, decoherence, or discontinuities. Yet, the benefit of such a marriage remains speculative. Second, incorporating extended Landau–Zener theories—most notably the Zhu–Nakamura model^80^ used in the “global-switching” surface hopping algorithm^81,82^ —could improve accuracy in the most demanding regimes, although this possibility has not been tested on the present systems. Third, the problems with discontinuities could be mitigated by calculating second derivatives of potential energies from forces and velocities as $\frac{d^{2}}{dt^{2}}\Delta E=-\frac{d}{dt}(\overset{\to }{v}\cdot \Delta \overset{\to }{F})$ . The advantage of such an approach comes from the fact that forces are typically less sensitive to orbital rotations than energies and do not exhibit such discontinuities.

Finally, we emphasize that all three surface–hopping variants investigated here deliver essentially comparable accuracy across the molecular test set, when the potential energy surfaces were reasonably continuous. The curvature-driven implementations, κFSSH and λFSSH, are attractive chiefly because they can be grafted onto existing FSSH codes with minimal effort. LZSH retains its appeal through conceptual transparency and an intrinsic immunity to the long-standing decoherence problem, whereas the canonical FSSH algorithm remains the most general and, in principle, systematically improvable framework. In day-to-day applications, however, the performance gap among the three approaches appears modest. As we, and many others, have repeatedly noted, the dominant source of uncertainty in computational photodynamics is the underlying electronic structure model; curvature-based schemes place even tighter demands on its fidelity.

In summary, unless a new curvature-based formulation demonstrably overcomes the structural issues identified here, LZSH, despite its 100 years of history, remains the most reliable NACV-free surface-hopping algorithm for molecular photodynamics.

## Supplementary Material



## Notes

Details on the vibronic potential of uracil, comparison of *ab initio* dynamics with and without the LZSH discontinuity patch, and hopping position analysis for a selected trivial crossing model (PDF)
