# Supplementary material for: Critical Assessment of Curvature-Driven Surface Hopping Algorithms
Source: J Chem Theory Comput. 2025 Sep 16;21(19):9784–98. doi: 10.1021/acs.jctc.5c01176 (PMC12529913; doi:10.1021/acs.jctc.5c01176)
Supplement: Supplementary file 1 [file ct5c01176_si_001.pdf]

Supporting information

Critical Assessment of Curvature-Driven Surface Hopping Algorithms

Tomáš Jíra, Jiří Janoš and Petr Slavíček*
University of Chemistry and Technology, 166 28 Prague 6, Czech Republic
* Corresponding author: Petr.Slavicek@vscht.cz

I. Uracil VC Model Constants

Below are the constants for an 8-dimensional uracil model described in Ref. 1.

Mode	$\kappa^{(0)}$	$\kappa^{(1)}$	$\kappa^{(2)}$	$\kappa^{(3)}$	$\lambda^{(02)}$	$\lambda^{(13)}$	$\gamma^{(0)}$	$\gamma^{(1)}$	$\gamma^{(2)}$	$\gamma^{(3)}$
ν_{18}	-0.02203	0.09074	0.02748	-0.04054	-0.03538	0.08077	0.01938	0.00694	-0.00294	0.00752
ν_{20}	-0.12147	0.05316	0.11233	0.00747	-0.02049		0.01489	0.00828	0.00183	0.00546
ν_{21}	-0.09468	0.04454	0.14539	0.00050		0.07284	0.00970	0.00096	-0.00114	0.01108

Table S1: Uracil VC model constants for ν_{18}, ν_{20} and ν_{21} modes using the harmonic potential.

Mode	d_0	a	q_0	e_0	$\lambda^{(02)}$	$\lambda^{(13)}$
ν_{25}						
(D_0)	4.80270	–0.13675	0.02883	–0.00007	0.00114	0.12606
(D_1)	74.15995	–0.03064	–1.34468	–0.12082		
(D_2)	90.76928	–0.03374	–0.29923	–0.00916		
(D_3)	20.56079	–0.08044	0.38841	–0.02071		
ν_{26}						
(D_0)	22.92802	0.07438	–0.32069	–0.01274	0.13035	0.14272
(D_1)	18.27440	0.07911	–0.01711	–0.00003		
(D_2)	9.46894	0.08653	0.37635	–0.01037		
(D_3)	65.09678	0.03660	1.66312	–0.25639		
ν_{24}						
(D_0)	41.89704	0.04719	0.81440	–0.06431		–0.01832
(D_1)	38.37122	0.05231	0.37488	–0.01505		
(D_2)	39.25691	0.05286	0.14859	–0.00244		
(D_3)	37.97847	0.05431	–0.18152	–0.00366		

Table S2: Uracil VC model constants for the ν_{25}, ν_{26} and ν_{24} modes using the Morse potential.

Mode	$k^{(0)}$	$k^{(1)}$	$k^{(2)}$	$\lambda^{(01)}$	$\lambda^{(12)}$
ν_{10}	0.03317	0.01157	0.01534	0.04633	0.03148
ν_{12}	0.02979	0.01488	0.01671	0.03540	0.03607

Table S3: Uracil VC model constants for ν_{10} and ν_{12} modes using the quartic potentials.

II. LZSH Discontinuity Patch Effect

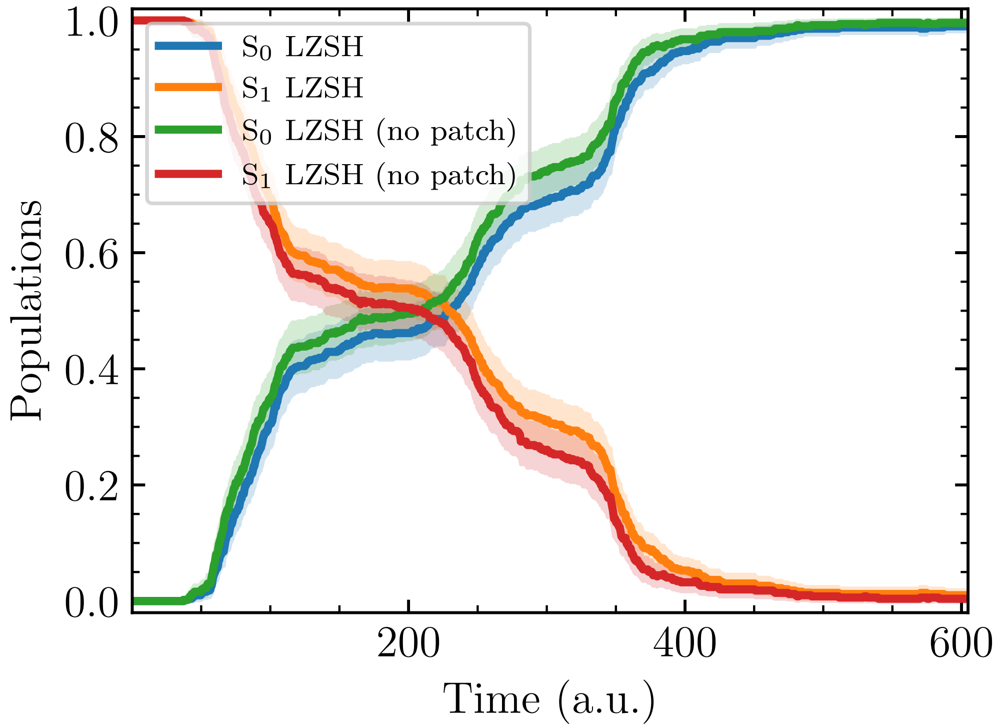

Figure S1: Comparison of electronic population dynamics in *cis*-stilbene using the LZSH method with and without the discontinuity correction described in the main manuscript.

III. Hopping Positions for κ/λFSSH for a Trivial Crossing Model

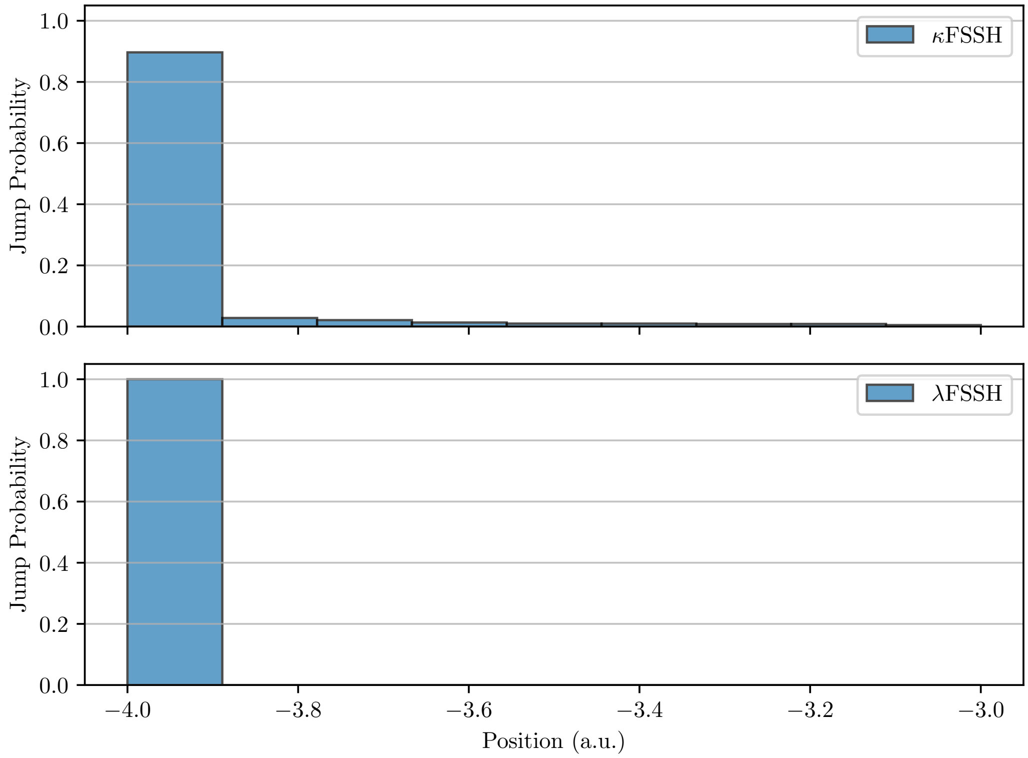

Figure S2: Histogram of hopping positions for the third model from Figure 4 (main text), comparing κFSSH and λFSSH. The discrepancy between the methods originates from the long-tailed behavior of the κTDC.

References

Patricia Vindel-Zandbergen, Spiridoula Matsika, and Neepa T. Maitra. Exact-factorization-based surface hopping for multistate dynamics. *J. Phys. Chem. Lett.*, 13(7):1785–1790, 2022. doi: 10.1021/acs.jpclett.1c04132.
